# A Study Assessing the Association of Glycated Hemoglobin A_1C_ (HbA_1C_) Associated Variants with HbA_1C_, Chronic Kidney Disease and Diabetic Retinopathy in Populations of Asian Ancestry

**DOI:** 10.1371/journal.pone.0079767

**Published:** 2013-11-07

**Authors:** Peng Chen, Rick Twee-Hee Ong, Wan-Ting Tay, Xueling Sim, Mohammad Ali, Haiyan Xu, Chen Suo, Jianjun Liu, Kee-Seng Chia, Eranga Vithana, Terri L. Young, Tin Aung, Wei-Yen Lim, Chiea-Chuen Khor, Ching-Yu Cheng, Tien-Yin Wong, Yik-Ying Teo, E-Shyong Tai

**Affiliations:** 1 Saw Swee Hock School of Public Health, National University of Singapore, Singapore, Singapore, Singapore; 2 Singapore Eye Research Institute, Singapore National Eye Centre, Singapore, Singapore, Singapore; 3 Department of Paediatrics, National University of Singapore, Singapore, Singapore, Singapore; 4 Life Sciences Institute, National University of Singapore, Singapore, Singapore; 5 Genome Institute of Singapore, Agency for Science, Technology and Research, Singapore, Singapore, Singapore; 6 Department of Ophthalmology, National University of Singapore, Singapore, Singapore, Singapore; 7 Centre for Eye Research Australia, University of Melbourne, Melbourne, Australia; 8 Department of Medicine, National University of Singapore, Singapore, Singapore, Singapore; 9 Department of Statistics and Applied Probability, National University of Singapore, Singapore, Singapore, Singapore; 10 Centre for Quantitative Medicine, Office of Clinical Sciences, Duke-NUS Graduate Medical School, Singapore, Singapore, Singapore; 11 Center for Human Genetics, Duke University Medical Center, Durham, North Carolina, United States of America; 12 Duke-NUS Graduate Medical School, Singapore, Singapore, Singapore; 13 NUS Graduate School for Integrative Science and Engineering, National University of Singapore, Singapore, Singapore, Singapore; Zhongshan Ophthalmic Center, China

## Abstract

Glycated hemoglobin A_1C_ (HbA_1C_) level is used as a diagnostic marker for diabetes mellitus and a predictor of diabetes associated complications. Genome-wide association studies have identified genetic variants associated with HbA_1C_ level. Most of these studies have been conducted in populations of European ancestry. Here we report the findings from a meta-analysis of genome-wide association studies of HbA_1C_ levels in 6,682 non-diabetic subjects of Chinese, Malay and South Asian ancestries. We also sought to examine the associations between HbA_1C_ associated SNPs and microvascular complications associated with diabetes mellitus, namely chronic kidney disease and retinopathy. A cluster of 6 SNPs on chromosome 17 showed an association with HbA_1C_ which achieved genome-wide significance in the Malays but not in Chinese and Asian Indians. No other variants achieved genome-wide significance in the individual studies or in the meta-analysis. When we investigated the reproducibility of the findings that emerged from the European studies, six loci out of fifteen were found to be associated with HbA_1C_ with effect sizes similar to those reported in the populations of European ancestry and P-value ≤ 0.05. No convincing associations with chronic kidney disease and retinopathy were identified in this study.

## Introduction

Glycated hemoglobin A_1C_ (HbA_1C_) is formed through the non-enzymatic glycation of hemoglobin. In patients with diabetes mellitus, it is used as a therapeutic target against which glucose lowering therapies can be titrated, as it shows a good correlation with the risk of developing microvascular complications associated with diabetes mellitus. It also shows less intra-individual variability than both fasting glucose and 2 hour post challenge glucose after an oral glucose tolerance test and can be measured without the need for fasting. For these reasons, it has been recommended as a criterion to diagnose diabetes [1,2].

HbA_1C_ levels show significant heritability, with twin studies conducted in Europeans estimating a heritability of 75% [3]. Several genome-wide association studies (GWAS) have been conducted in populations of European ancestry: a GWAS conducted in 14,618 women identified 4 loci harboring variants associated with HbA_1C_ [4]; a study of 1,782 Europeans found suggestive evidence for an association with HbA_1C_ at the *TCF7L2* locus [5]; and a meta-analysis of 31 genome-wide studies across 68,000 European samples revealed 10 loci harboring variants associated with HbA_1C_ [6]. However, several questions remain unanswered.

Firstly, are there novel variants that can be found through GWAS in populations of non-European ancestry, either because the variants are more common in those populations, or there are ethnic specific effects observed? Secondly, are the findings from studies conducted in European populations transferable to Asian populations? A study in the United States showed differences in both allele frequency and the magnitude of the association for HbA_1C_ associated SNPs between race/ethnic groups [7]. However, few studies have been conducted in Asians with the exception of one study conducted in over 8,000 Koreans which identified variants at the *CDKAL1* locus which were associated with type 2 diabetes (T2D) and HbA_1C_ [8]. 

Thirdly, it is also unclear if these variants are associated with the complications associated with T2D. One study conducted in the North America showed that HbA_1C_ associated SNPs were not associated with mortality [7] and coronary artery disease [6]. However, to our knowledge, the association between HbA_1C_ associated SNPs and microvascular complications (which show a much stronger association with blood glucose than either mortality or cardiovascular disease) has not been examined. The relationship between glycemia and microvascular complications in patients without T2D may be particularly relevant given that we have shown that the relationships between blood glucose and both macro- and micro-vascular disease are monotonic, and extend down into the normal blood glucose range [9-11].

Here we describe the findings of three genome-wide surveys of HbA_1C_ that were conducted in 6,682 individuals from three populations of Asian ancestry, which include 3,427 Chinese, 1,735 Malays and 1,520 Asian Indians residing in Singapore. We aimed to answer the three questions mentioned above.

## Materials and Methods

### Ethics statement

The sample recruitment was approved by The Singapore General Hospital Ethics Committee and the Singapore Eye Research Institute Ethics Committee. The genetic analysis was approved by the National University of Singapore Institutional Review Board (Approval Certificate NUS465). All participants provided written informed consents.

### Study cohorts

A flow chart which described the process from sample recruitment to association analysis was presented in Figure S1.

The Singapore Prospective Study Program (SP2) first surveyed the subjects between the ages of 24 and 95 years from four previous cross-sectional studies: Thyroid and Heart Study (1982–1984), National Health Survey (1992), National University of Singapore Heart Study (1993–1995) and the National Health Survey (1998). Each of these studies randomly sampled individuals from the Singapore population, although a disproportionate sampling scheme was utilized to increase the sample sizes of the minority ethnic groups (Malays and Asian Indians). Between 2003 and 2007, we revisited these subjects. Of the 10,747 subjects who were eligible, we successfully recruited 6,968 subjects who completed a questionnaire. 5,157 subjects in this cohort also completed the subsequent clinical examination which included venesection for the collection of, among other things, DNA for genetic analysis. The Singapore Malay Eye Study (SiMES) is a population-based, cross-sectional epidemiological study of 3,280 Malay adults living in 15 residential districts in the southwestern part of Singapore [12]. The Singapore Indian Eye Study (SINDI) and the Singapore Chinese Eye Study (SCES) are respectively the Asian Indian and Chinese equivalent to SiMES, where the sampling was similarly performed in the same 15 residential districts and included 3,400 Asian Indians and 3,353 Chinese respectively. 

### Genotyping and data quality control

Of the 5,157 SP2 individuals who finished the questionnaire and clinical examination, 3,236 individuals of Chinese ancestry had their blood samples collected. We genotyped 2,857 of these individuals on three different Illumina arrays (San Diego, California, the United States), with 583 samples on the HumanHap550v3, 1,467 samples on the HumanHap 610Quad and 1,016 samples on the HumanHap 1Mduov3. The coverage of these SNP arrays in HapMap JPT+CHB panel were comparable to that in populatoins of European ancestry [13,14]. Samples from the remaining three cohorts, 1,952 from SCES, 3,072 from SiMES and 2,953 from SINDI, were successfully genotyped on the HumanHap 610Quad array. For each cohort, a preliminary SNP quality control (QC) was conducted to remove SNPs that: (i) are monomorphic; (ii) are of high rates of missingness (> 5%); or (iii) exhibit evidence of departure from Hardy-Weinberg Equlibrium (HWE *P* < 10^-6^); (iv) cannot be mapped to the forward strand of the human genome build 36. Using the remaining SNPs, samples are removed on the basis of missingness, excessive heterozygosity, relatedness, discordant ethnic membership or discrepancy between the genetically inferred and clinically recorded genders. With the post-QC samples, we re-analyzed the SNP quality metrics. We excluded a SNP if it: (i) could not be mapped to the forward strand of the human genome build 36; (ii) displayed excessive missingness (>5%); (iii) exhibited evidence of departure from Hardy-Weinberg Equlibrium (HWE *P* ≤ 10^-6^); (iv) was monomorphic; or (v) was non-autosomal. The numbers of samples removed in each step and remained after QC of our studies were presented in Figure S1.

### Imputation

To maximize the information available, we imputed the genotypes for all samples using the phased haplotypes in Phase 2 release 22 of the International HapMap Project (HapMap2 r22) using either IMPUTE v0.5.0 [15] (SP2, SiMES and SINDI) or IMPUTE v2.1.2 (SCES) [16]. The two IMPUTE versions implemented different algorithms, but the imputation accuracy does not differ significantly [16]. The Chinese samples in SP2 and SCES were imputed using reference panels derived from HapMap Han Chinese from Beijing (CHB) and the Japanese in Tokyo (JPT) resulting about 2.4 million imputed SNPs. The Malay and Indian samples were imputed against a combined reference panel of Caucasians (CEU), East Asians (JPT+CHB) and Africans (YRI) resulting in about 1.9 million imputed SNPs. Each chromosome was divided into 5Mb regions for imputation with a 250kb buffer added to each flank. We retained only the imputed SNPs with information criterion ≥ 0.5.

### Phenotype definition

HbA_1C_ was measured by high pressure liquid chromatography using a method that was certified by the National Glyoprotein Standardization program with controls traceable to the diabetes control and complications trial [10,11]. 

Creatinine was measured by enzymatic methods implemented on the ADVIA 2400 (Bayer Diagnostics, Tarrytown, New York, United States of America) [17]. Glomerular filtration rate (GFR) was estimated using the Modification of Diet in Renal Disease (MDRD) [18]. Chronic kidney disease (CKD) was defined as estimated glomerular filtration rate (eGFR) less than 60 and greater than or equal to 0, while control’s eGFR is greater than or equal to 60 [11]. 

Diabetic retinopathy (DR) was evaluated following a standard protocol based on retinal photographs which were graded according to a modified scale from Airlie House classification system [19]. Two definitions for DR were employed, (i) moderate/severe DR was defined as grade ≥ 30 (ii) any DR was defined as grade ≥ 14. In both instances, controls had grade < 14 [20-22].

### Associations with HbA_1C_

To identify SNPs associated with HbA_1C_, we excluded all subjects with diabetes which was defined as anyone who a) gave a self-reported a diagnosis of diabetes given by a physician, b) was taking medications for diabetes, or c) had HbA_1C_ ≥ 6.5%. In addition, in SP2, we also excluded individuals with fasting glucose ≥ 7mmol/L [2]. More detail on the number of excluded samples can be found in Figure S1.

The three ethnic groups in Singapore exhibit significant genetic differences [23] and phenotypic differences [24,25]. In order to avoid any population stratification that might occur due to these differences, association testing was first performed within each ethnic group. For each cohort, the HbA_1C_ percentage was regressed against the allele dosage of the SNP assuming a linear model adjusted for age and gender using SNPTEST v2.2 [15]. We further controlled for population stratification by adjusting for principal components (PCs). The principal component analyses was calculated using an independent set of SNPs (r^2^<0.2) across the autosomal chromosomes by EIGENSTRAT [26]. The distribution of the PCs was observed in each cohort. For SiMES, the first two principal components from the PCA were additionally included as covariates to control for population stratification, while three principal components were included for SINDI. No principal component was adjusted for in the Chinese studies because there is no significant stratification shown in the cohorts (Figure S2, S3, S4). We further removed the genotyped and imputed SNPs of minor allele frequency (MAF) < 1%, missing rate > 5% or HWE P value ≤ 10^-6^. Finally, we applied genomic control to each study before meta-analysis to control for the remaining minor inflation in the test statistics. The inflation factor (λ_GC_) calculated using the genomic control approach was used to estimate the extent to which the P-values are skewed by the inflation factors [27]. We used the fixed-effect inverse variance model in METAL to perform the meta-analyses [28]. The four Chinese GWAS (SP2-550, SP2-610, SP2-1M, SCES) were first meta-analyzed together to yield a single study for the Chinese ethnic group. Subsequently, a meta-analysis was performed to integrate the results from the combined Chinese study, SiMES and SINDI. 

### Selection of European established index SNPs associated with HbA_1C_

We searched the literature to identify SNPs associated with HbA_1C_ which had shown strong evidence of association with HbA_1C_ in populations of European ancestry. We identified 11 SNPs from Soranzo et. al. [6], 4 SNPs from Pare et. al.[4], 1 SNP from Franklin et. al. [5] and investigated the transferability of these 16 associations to the Asian populations that are the subject of this study.

### Calculation of regional F_ST_ and haplotype entropy

The fixation index (F_ST_) is a common measure of differentiation and genetic distance. It has been shown that the F_ST_ scores for SNPs in a region are indicative of the reproducibility of association signals across populations [29]. We thus calculated the regional F_ST_ surrounding an index SNP, defined as the average of SNP-level F_ST_ for SNPs that are shared across the populations and are located within 50 Kb of either flanks of the index SNP. It has also been shown that evaluating the entropy of haplotype frequencies can be predictive of meta-analysis efficiency, which is a surrogate for the transferability of genetic association signals across populations [29,30]. For each index SNP, we also considered the 100 Kb region flanking the index SNP across the populations, and identified the set of unique haplotypes that are present in at least 2% of each population. These haplotypes were collated across every pair of populations consisting of CEU and one of the three Singapore populations, and the frequency of each of the *i* ^th^ haplotype in the *j* ^th^ population was calculated and defined as *f*_*ij*_. The posterior probability of each population conditional on the *i* ^th^ haplotype, *F*_*ij*_, is *f*_*ij*_/(Σ_*j*_ *f*_*ij*_), and the relative mutual information for the *i*th haplotype can be calculated as RMI(*i*) = 1 + Σ_j_(*F*_*ij*_ log *F*_*ij*_)/log(2). We thus defined the haplotype entropy for the genomic region as the overall-frequency weighted sum of the individual relative mutual information estimates, or Σ_i_[(Σ_i_ *f*_i•_) RMI(*i*)]/ (Σ_i_ *f*_i•_), where *f*_i•_ represent the frequency of the *i* ^th^ haplotype in the two populations. The haplotype entropy metric effectively quantifies the degree of population-specificity of each haplotype, and is bounded between 0 and 1. Larger values mean there are particular haplotypes that are more common in some populations than others, and thus indicate that there is greater haplotype diversity between the populations queried. The significance of the difference in regional F_ST_ and haplotype entropy between failed and replicated associations was evaluated by one-tail t-tests assuming equal variance.

### Associations with CKD and DR

These associations were assessed separately in those with and without T2D. For these analyses, T2D cases were defined as HbA_1C_ ≥ 6.5%, or self-reported diabetic history. T2D controls were defined as HbA_1C_ < 6% and no diabetes history. Especially for SP2 samples, T2D controls were defined as fasting glucose ≤ 6 mmol/L and no diabetic history. We did not conduct the association tests of CKD and DR in the SP2 individuals with diabetes due to the very limited number of both CKD cases and DR cases in this cohort (data not shown). 

Association tests were done using logistic regression implemented with SNPTEST v2.2 without considering any covariates except PC1 and PC2 for SiMES and PC1 to PC3 for SINDI. We tested the association of the 16 European established SNPs with DR and CKD in our cohorts. The association was stratified into T2D cases and T2D controls, and the association results were meta-analyzed within cases, controls and all samples separately using the fixed effect inverse variance scheme as implemented in METAL. 

## Results

### Genome-wide association study and meta-analysis of HbA_1C_

The characteristics of the samples from the investigated studies are summarized in Table 1. The inflation factors for all 3 populations were all close to 1, suggesting that significant population stratification was not present or properly controlled in our study. Moreover, the meta-analysis inflation factor was 0.999.

**Table 1:** Demographics of the investigated studies.

Study	SP2-1M	SP2-610	SP2-550	SCES	Chinese combine	SiMES	SINDI
N	779	797	266	1585	3427	1735	1520
Men (%)	61.54	16.99	66.03	50.38	46.35	49.74	49.14
age	46.74(10.13)	47.42(10.61)	48.27(12.24)	57.69(9.40)	52.08(11.37)	57.59(11.21)	56.42(9.76)
BMI	22.75(3.39)	22.20(3.71)	23.28(3.47)	23.46(3.46)	22.99(3.54)	25.73(5.09)	25.66(4.52)
HbA1c (%)	5.6(0.4)	5.63(0.4)	5.60(0.4)	5.81(0.3)	5.71(0.4)	5.71(0.4)	5.68(0.4)
HbA1c (mmol/mol)	38(4.4)	38(4.4)	38(4.4)	40(3.3)	39(4.4)	39(4.4)	39(4.4)
Inflation Factor	1.01	1.02	0.994	0.997	1.005	1.003	0.998

N, sample size; Men (%) is the percentage of males in each cohort.

The inflation factors were obtained after adjusting for age, sex, BMI and PCs (for SiMES and SINDI only).

Age, BMI and HbA_1C_ are given as mean(SD). HbA_1C_ is given in both DCCT-derived (%) and IFCC-recommended (mmol/mol) units.

SP2, Singapore Prospective Study Program. SP2-1M, SP2-610 and SP2-550 are the SP2 samples genotyped on Ilumina HumanHap 1M Duo, 610 Quad and 550 v3 SNP arrays respectively.

SCES, Singapore Chinese Eye Study. The combined Chinese included the three SP2 cohorts and SCES. SiMES, Singapore Malay Eye Study.

SINDI, Singapore Indian Eye Study.

**Figure 1:**
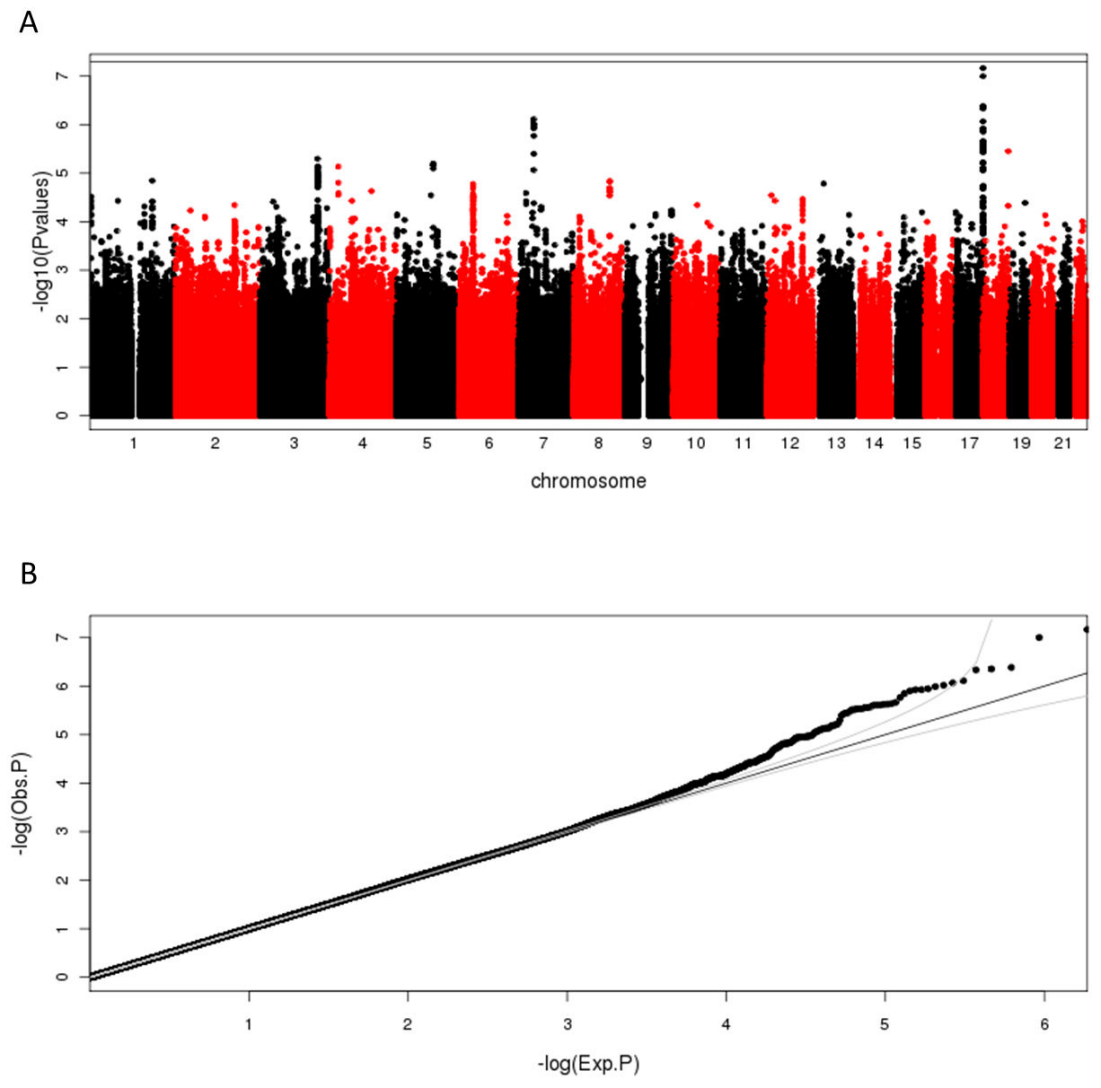
Manhattan plot and Q-Q plot of genome-wide meta-analysis. (A) The Manhattan plot of the meta-analysis of Chinese, Malays and Indians. The minus log_10_ of P-values (Y-axis) of inverse-variance meta-analysis across the 22 autosomal chromosomes after genomic control were plotted against the genomic coordinates. The horizontal line represents the genome-wide significant level of 5x10^-8^. (B) Q-Q plot of the observed P-values (Y-axis) against the expected P-values (X-axis). The diagonal line and its 95% confidence envelop were also plotted.

The separate genome-wide scans for Chinese, and Indians did not reveal any genome-wide significant associations (Figure S5A and 5C). In the Malays, six SNPs at one locus near the *G6PC3* gene on chromosome 17 exhibited evidence in excess of genome-wide significance (leading SNP: rs12603404, *P* = 3.89x10^-22^, Figure S5B). However, this association was not replicated in the Chinese or the Indians (Table S1) despite similar allele frequencies for these SNPs in all 3 populations. The meta-analysis of all three studies across a total of 6,682 samples did not reveal any additional SNPs at genome-wide significance (Figure 1). The quantile-quantile plots (Q-Q plots) adjusted for the corresponding inflation factors did not show significant inflation, nor did they show obvious departure from the null hypothesis of no association between genes and HbA_1C_ in our cohorts, except for the Malays (Figure S6). 

#### Transferability of European findings in Asian populations

We next sought to determine whether the previous findings established in populations of European ancestry are relevant and transferable to populations of non-European ancestry. Of the 16 SNPs that show an association with HbA_1C_ in populations of European Ancestry, 14 were present and polymorphic in our studies across all three ethnic groups. For the remaining two index SNPs, rs16926246 at the *HK1* locus is present only in the Indian GWAS because of the poor imputation quality in Chinese and Malays and rs1800562 at the *HFE* locus had a MAF of 0.2% in Indians and is monomorphic in both Chinese and Malays, and thus did not pass our quality control filter. As a result, we have 14 SNPs in Chinese and Malays, 15 SNPs in Indian and 14 SNPs in meta-analysis respectively for our transferability investigation.

**Figure 2:**
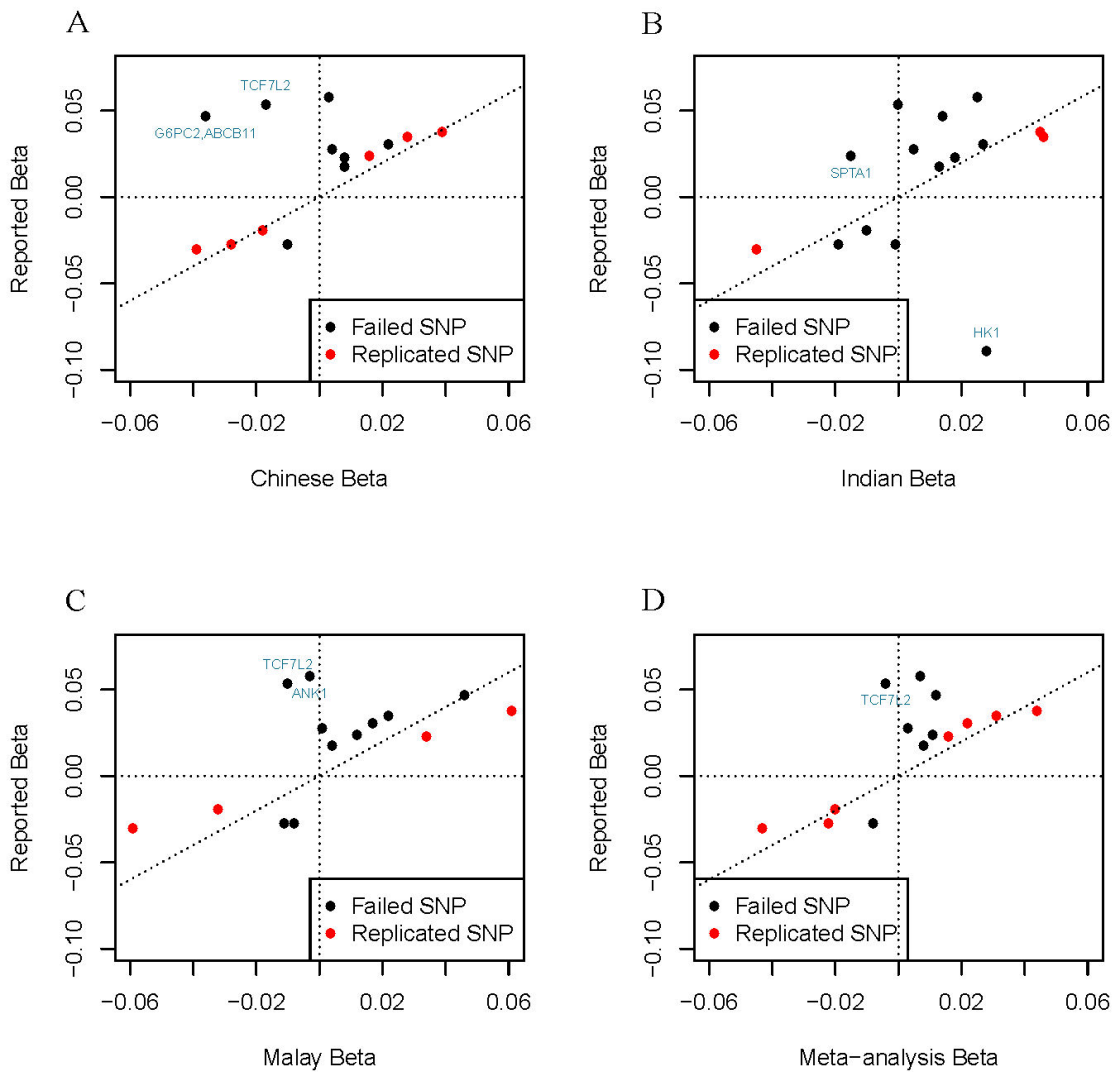
Bivariate plots of the effect sizes in Asian cohorts and European ancestry cohorts. The effect sizes reported in the European ancestry were plotted against the ones discovered in our studies. The Y-axis corresponds to the European effect size, while the X-axis corresponds to the effect in Chinese (A), Indians (B), Malays (C) and Meta-analysis (D). The SNPs which are replicated in our studies at the P-value of 0.05 level were designated by red dots, while the SNPs failed to be replicated were designated by black dots. The SNPs with inconsistent effect directions between European and each population were labeled by the reported gene names.

Six, four, three and seven of the SNPs under investigation were associated with HbA_1C_ variation in the Chinese, Malays, Asian Indians and the combined meta-analysis respectively at a P-value ≤ 0.05 (Table 2). To determine the reasons for the failure of other SNPs to show even nominal association with HbA_1C_, we next estimated the powers of our study to detect these associations based on the sample sizes available to us, the allele frequency of the variants in our populations and the effect estimates derived from the respective populations (Table S2). We have higher powers at the SNPs with P-value ≤ 0.05 (range from 17.1% to 93.8% in the individual ethnic groups) than the SNPs with P-value > 0.05 (range from 5.0% to 36.7% in the individual ethnic groups). Given that our study was underpowered to detect many of these associations, we also compared the effect sizes for each of these SNPs in our study and those observed in populations of European ancestry (Figure 2). For most of the variants, including those that failed to replicate in our studies, the directions of the effects in our study were consistent with those seen in the populations of European ancestry. In the meta-analysis across all three ethnic groups, there was only one SNP, rs7903146 in *TCF7L2*, which displayed an opposite direction of effect compared to that observed in populations of European ancestry. However, it should be noted that the allele frequency and sample size resulted in very limited power (6.2% in the meta-analysis) to detect the association at this locus in our study.

**Table 2:** Associations with HbA1c of the European established index SNPs in our cohorts.

					EAF	Beta		Chinese				Malay				Indian				Meta-analysis		
SNP	Chr	BP	Gene	EA	(European)	(European)		EAF	Beta	P-value		EAF	Beta	P-value		EAF	Beta	P-value		EAF	Beta	P-value
rs2779116	1	156,852,039	SPTA1	T	0.27	0.024		0.43	0.016	**3.72E-02**		0.46	0.012	3.40E-01		0.16	-0.015	3.72E-01		0.40	0.011	6.99E-02
rs1402837	2	169,465,600	G6PC2	T	0.23	0.023		0.41	0.008	3.11E-01		0.38	0.034	**8.05E-03**		0.22	0.018	2.28E-01		0.37	0.016	**1.10E-02**
rs552976	2	169,499,684	G6PC2,ABCB11	G	0.64	0.047		0.99	-0.036	3.24E-01		0.97	0.046	2.00E-01		0.83	0.014	3.88E-01		0.87	0.012	3.96E-01
rs730497	7	44,190,246	GCK	G	0.83	-0.030		0.80	-0.039	**8.57E-05**		0.88	-0.059	**2.97E-03**		0.88	-0.045	**2.05E-02**		0.83	-0.043	**8.14E-08**
rs1799884	7	44,195,593	GCK	T	0.18	0.038		0.20	0.039	**7.49E-05**		0.11	0.061	**2.16E-03**		0.12	0.045	**2.09E-02**		0.17	0.044	**5.62E-08**
rs6474359	8	41,668,351	ANK1	T	0.97	0.058		0.97	0.003	8.80E-01		0.98	-0.003	9.53E-01		0.97	0.025	5.59E-01		0.97	0.007	7.23E-01
rs4737009	8	41,749,562	ANK1	G	0.76	-0.027		0.51	-0.010	3.11E-01		0.60	-0.011	5.03E-01		0.78	-0.001	9.42E-01		0.58	-0.008	2.59E-01
rs13266634	8	118,253,964	SLC30A8	T	0.30	-0.019		0.47	-0.018	**2.13E-02**		0.43	-0.032	**1.23E-02**		0.23	-0.010	4.91E-01		0.42	-0.020	**1.03E-03**
rs7072268	10	70,769,919	HK1	T	0.50	0.018		0.76	0.008	3.71E-01		0.71	0.004	7.75E-01		0.43	0.013	3.13E-01		0.66	0.008	1.94E-01
rs7903146	10	114,748,339	TCF7L2	T	0.28	0.054		0.02	-0.017	5.34E-01		0.04	-0.010	7.71E-01		0.27	0.000	9.98E-01		0.19	-0.004	7.11E-01
rs1387153	11	92,313,476	MTNR1B	T	0.28	0.028		0.47	0.004	6.56E-01		0.42	0.001	9.56E-01		0.37	0.005	7.15E-01		0.44	0.003	5.94E-01
rs7998202	13	112,379,869	ATP11A,TUBGCP3	G	0.14	0.031		0.06	0.022	1.71E-01		0.08	0.017	4.67E-01		0.09	0.027	2.13E-01		0.08	0.022	**4.93E-02**
rs1046896	17	78,278,822	FN 3K	T	0.31	0.035		0.51	0.028	**3.20E-04**		0.46	0.022	8.05E-02		0.37	0.046	**4.05E-04**		0.47	0.031	**2.41E-07**
rs855791	22	35,792,882	TMPRSS6	G	0.58	-0.027		0.46	-0.028	**3.82E-04**		0.43	-0.008	5.22E-01		0.50	-0.019	1.21E-01		0.46	-0.022	**2.35E-04**
rs16926246	10	70,763,398	HK1	T	0.10	-0.089		-	-	-		-	-	-		0.05	0.028	3.96E-01		-	-	-

Chr, represents the chromosome number of the SNP; BP, base pair position; EA, effect allele; N, sample size; EAF, effect allele frequency; Beta, lineaer regression coefficient; SE, standard error of Beta. Significant P-values less than or equal to 0.05 were highlighted in bold.

Given the varying success in the Asian studies at reproducing the reported associations, we also investigated whether population differences in the genetic architecture could have contributed to this failure to replicate the associations. We calculated the regional F_ST_ and haplotype entropy between the HapMap Europeans (CEU) and each of the three Asian populations in the Singapore Genome Variation Project (SGVP) [23] for a 100 Kb region flanking each of the 16 index SNPs (Table S2). SGVP consists of three panels, Singapore Chinese (CHS), Malays (MAS) and Indians (INS). We only considered the pairs of CEU and SGVP populations as we are interested to evaluate the replicability of findings that were originally discovered in populations of European ancestry. We observed that, across our genome-wide surveys, the regional F_ST_ for the loci that replicated were significantly lower than those that failed to replicate (two-sample t-test P-value = 3.12x10^-5^), indicating that genetic diversity between European and the Asian populations is likely to contribute to our inability to reproduce the associations at the index SNPs. The haplotype entropy values were also lower for the loci that successfully replicated than those that did not replicate but this difference did not reach statistical significance (P-value = 0.08). 

### Associations with diabetic retinopathy and chronic kidney disease

The case-control studies of CKD and DR were done in over 1800 subjects with T2D and 4400 without T2D. The prevalence of both CKD and DR are much higher in T2D cases than in T2D controls (Table 3). No SNP was associated with CKD in subjects without T2D. Of the 15 HbA_1C_ index SNPs, one SNP, rs2779116 at the *SPTA1* locus, was significantly associated with CKD in subjects with T2D (P value = 1.7 x 10^-3^, OR = 1.31). The significance was diminished after combining with the results from those without T2D (Table 4). Two other SNPs (near the *ANK1* and *HK1* loci) also showed nominal significance in the meta-analysis combining those with and without T2D. However, neither of them reached the Bonferroni corrected significance level of 8.3 x 10^-4^ (0.05 divided by 15 SNPs and 4 phenotypes). We observed only one SNP, rs16926246 at the *HK1* locus, which showed nominal significant association with moderate/severe diabetic retinopathy (P value = 3.88 x 10^-2^, OR = 0.54) when all subjects with and without T2D were combined (Table S3). Moreover, for any retinopathy, there were several nominally significant associations with SNPs close to the *ANK1*, *SLC30A8*, *MTNR1B*, *TMPRSS6* and *HK1* loci. However, none remained after Bonferroni correction (Table S4). Most of these were observed in subjects without T2D.

**Table 3:** Sample size and prevalence rate of stratified cohorts in CKD and DR analysis.

	CKD	Moderate/ severe DR	Any DR
**T2D cases**	**2072, 22.1%**	**1802, 21.0%**	**2022, 29.6%**
SCES	302(42/260)	267(46/221)	296(75/221)
SiMES	794(279/515)	677(130/547)	761(214/547)
SINDI	976(136/840)	858(203/655)	965(310/655)
**T2D controls**	**5201, 6.9%**	**4409, 0.4%**	**4619, 4.9%**
SCES	1090(51/1039)	1035(2/1033)	1082(49/1033)
SP2-1M	891(30/861)	609(5/604)	641(37/604)
SP2-610	811(20/791)	529(3/526)	549(23/526)
SiMES	1240(205/1035)	1130(3/1127)	1195(68/1127)
SINDI	1169(52/1117)	1106(5/1101)	1152(51/1101)
**Total**	**7273**	**6211**	**6641**

Sample sizes of separate cohorts are represented by # total (# cases / # controls)

For the stratified totals, "T2D cases" and "T2D controls", the number of samples and prevalence rate of CKD and DR are present.

The CKD case was defined as 0 ≤ eGFR < 60. The CKD control was defined as eGFR ≥ 60;

Retinopathy was graded according to the modified Airlie House classification system. The moderate/severe DR case was defined as grade ≥ 30, while any DR was defined as grade ≥ 14. In both definitions, controls were those of grade < 14.

**Table 4:** Association evidence of European established HbA_1C_ SNPs with CKD.

							Combined			T2D Cases			T2D Controls	
SNP	Chr	BP	Gene	EA	OA	EAF	OR[0.95CI]	P-value		OR[0.95CI]	P-value		OR[0.95CI]	P-value
rs2779116	1	156,852,039	SPTA1	T	C	0.42	1.16 [1.03, 1.31]	**1.24E-02**		1.31 [1.11, 1.56]	**1.70E-03**		1.04 [0.88, 1.22]	6.48E-01
rs1402837	2	169,465,600	G6PC2	T	C	0.36	1.04 [0.92, 1.16]	5.40E-01		1.02 [0.86, 1.20]	8.35E-01		1.06 [0.90, 1.24]	5.11E-01
rs552976	2	169,499,684	G6PC2,ABCB11	A	G	0.10	0.96 [0.77, 1.20]	7.29E-01		0.81 [0.61, 1.07]	1.42E-01		1.29 [0.89, 1.87]	1.78E-01
rs730497	7	44,190,246	GCK	A	G	0.15	0.94 [0.80, 1.10]	4.48E-01		0.96 [0.77, 1.21]	7.48E-01		0.91 [0.73, 1.15]	4.49E-01
rs1799884	7	44,195,593	GCK	T	C	0.14	0.94 [0.80, 1.11]	4.71E-01		0.97 [0.77, 1.22]	7.91E-01		0.91 [0.72, 1.15]	4.47E-01
rs6474359	8	41,668,351	ANK1	T	C	0.97	1.60 [1.07, 2.38]	**2.19E-02**		1.53 [0.84, 2.80]	1.69E-01		1.65 [0.97, 2.82]	6.52E-02
rs4737009	8	41,749,562	ANK1	A	G	0.41	0.90 [0.78, 1.04]	1.44E-01		0.84 [0.68, 1.03]	8.58E-02		0.96 [0.79, 1.17]	7.01E-01
rs13266634	8	118,253,964	SLC30A8	T	C	0.42	1.03 [0.92, 1.15]	6.57E-01		1.04 [0.88, 1.22]	6.68E-01		1.02 [0.87, 1.19]	8.40E-01
rs7072268	10	70,769,919	HK1	T	C	0.67	1.17 [1.03, 1.31]	**1.15E-02**		1.18 [1.00, 1.39]	5.17E-02		1.15 [0.97, 1.37]	1.04E-01
rs7903146	10	114,748,339	TCF7L2	T	C	0.16	1.02 [0.85, 1.24]	7.99E-01		1.05 [0.84, 1.33]	6.59E-01		0.97 [0.70, 1.34]	8.59E-01
rs1387153	11	92,313,476	MTNR1B	T	C	0.43	0.93 [0.83, 1.04]	2.05E-01		0.92 [0.79, 1.07]	2.61E-01		0.95 [0.81, 1.11]	5.10E-01
rs7998202	13	112,379,869	ATP11A,TUBGCP3	A	G	0.92	0.97 [0.79, 1.18]	7.64E-01		0.87 [0.67, 1.13]	2.94E-01		1.12 [0.83, 1.52]	4.56E-01
rs1046896	17	78,278,822	FN 3K	**T**	C	0.45	1.05 [0.94, 1.17]	4.00E-01		1.07 [0.92, 1.25]	3.76E-01		1.02 [0.87, 1.20]	7.70E-01
rs855791	22	35,792,882	TMPRSS6	A	G	0.55	0.93 [0.83, 1.04]	1.88E-01		0.92 [0.79, 1.06]	2.51E-01		0.94 [0.81, 1.11]	4.78E-01
rs16926246	10	70,763,398	HK1	T	C	0.05	0.84 [0.47, 1.50]	5.53E-01		0.54 [0.27, 1.09]	8.53E-02		2.37 [0.81, 6.97]	1.17E-01

The association of CKD was done in T2D cases and controls separately. The results were then combined using fixed effect meta-analysis. Chr, represents the chromosome number of the SNPs; BP, base pair position; EA, effect allele; N, sample size; EAF, effect allele frequency. OR and 0.95 confident interval were calculated from the logistic regression coefficient and its standard error. Significant P-values less than or equal to 0.05 are highlighted in bold.

## Discussion

We have performed a genome-wide survey of HbA_1C_ variants in 6,682 Chinese, Malays and Asian Indians in Singapore. To the best of our knowledge, this is the first time a GWAS for HbA_1C_ has been reported for Malays and Indians. While a locus on chromosome 7 near the *G6PC3* locus reached genome-wide significance in Malays, this finding was not replicated in Chinese or Indians. The meta-analysis of these three ethnic groups also did not identify any associations that achieved genome-wide significance.

Our study confirms that genetic loci that have been previously established to be associated with HbA_1C_ in populations of European ancestry show similar direction and magnitude of effect in these Asian populations. Only a few of these associations were statistically significant, which likely relates to relatively low power in our study. For example, the frequency of the effect allele at the European index SNP (rs7903146) at *TCF7L2* is 28%, as compared to 2% and 4% in the Chinese and Malays respectively. Notably, this index SNP showed opposite effect directions across the three Asian studies. In addition, *TCF7L2* has been reported to be differentially selected between Asian and European populations [31]. 

Owing to the reliance of GWAS on tagging markers that, by themselves, are not functional, we also considered the possibility that differences in genetic architecture (measured using the F_ST_ score and haplotype entropy) at these established loci could contribute to the lack of transferability of the findings related to the index SNP between populations. Higher regional F_ST_ and haplotype entropy between Europeans and Chinese/Malays at loci such as *TCF7L2*, *HK1* and *G6PC2/ABCB11* suggest that the power to reproduce the European findings for these index SNPs would be even lower in our study populations than the allele frequency and sample size might suggest. 

A study conducted in over 8,000 Korean healthy individuals identified a SNP at the *CDKAL1* locus that has been consistently reported for T2D association across populations of varying ancestries [32-37] was also associated with HbA_1C_ [8]. However, the index SNP in this Korean study (rs7747752) did not show a statistically significant association with HbA_1C_ in our cohorts (P-value = 0.79 in Chinese, 0.92 in Malays, 0.19 in Indians, and 0.47 in meta-analysis). Nonetheless, this SNP shows an association with T2D in our populations that is similar to that observed in others [38] and the lack of association if our study is possibly due to the insufficient power (5.5% in Chinese, 5.1% in Malays, 25.1% in Indians and 10.2% in meta-analysis). 

In thinking about the associations between HbA_1C_ associated SNPs and DR/CKD, we need to consider the mechanisms that explain the association between these variants and HbA_1C_. The proportion of HbA_1C_ is highly correlated with the average glucose level in the blood over a period of time [39]. It would not, therefore, be surprising that several of the genetic loci identified in association with HbA_1C_ could show an association with microvascular complications that is mediated by blood glucose. Our study showed nominal associations between variants at the *SLC30A8* and *MTNR1B* loci with any diabetic retinopathy (Table S4). Since this phenotype is known to be associated with blood glucose [9-11], and both these loci are known to be associated with T2D and blood glucose, these findings are not surprising. On the other hand, several loci also showed associations with various erythrocyte parameters (*SPTA1*, *HFE*, *ANK1*, *HK1*, *TMPRSS6*). Furthermore, rare variants at these loci are known to cause hereditary anemia, suggesting that these variants primarily alter erythrocyte physiology. While these variants may affect the utility of HbA_1C_ in diabetes diagnosis, they are less likely to be associated with microvascular complications. Yet, we did observe an association between the index SNP at the *SPTA1* and CKD. In addition, variants at the *HK1* locus were associated with CKD, moderate/severe DR and any DR. An index SNP at the *ANK1* locus also associated with CKD and retinopathy in non-diabetic individuals. This raises the possibility that the pathogenesis of diabetic complications is more complex than that of blood glucose alone, and we should not ignore variants just because they are associated with erythrocyte parameters. However, it should be noted that most of these associations, apart from *SPTA1* with CKD, did not survive correction for multiple testing and the significance of these findings is uncertain.

Finally, there are some variants that do not show association with either blood glucose or erythrocyte parameters. These include FN *3K*, which deglycates hemoglobin and other glycated proteins formed under hyperglycemic conditions. These variants could potentially affect not only HbA_1C_, but also the downstream products of non-enzymatic protein glycation, the advanced glycation end-products, which may have an important role in the pathogenesis of microvascular complications associated with T2D [40]. In fact, a small study suggested that variants at this locus are associated with the progression of diabetic nephropathy [41]. Our data does not support this previous finding.

In conclusion, we identified a low frequency variant near the *G6PC3* locus in Malays living in Singapore, which was not replicated in Chinese and South Asians. This finding requires confirmation in future studies. We also showed that most SNPs associated with HbA_1C_ in populations of European ancestry are also relevant to Asian populations. Furthermore, among the 15 HbA_1C_ index SNPs, we identified one significant association between *SPTA1* and CKD.

## Supporting Information

Figure S1 **Flow chart of the GWAS meta-analysis.** SP2, Singapore Prospective Study Program; SCES, Singapore Chinese Eye Study; SiMES, Singapore Malay Eye Study; SINDI, Singapore Indian Eye Study; Illu610, Illu1M and Illu550 are Illumina HumanHap 610 Quad, 1M Duo and 550 v3 array platforms respectively.
(JPG)

Figure S2 **Principle component analysis of SP2 and SCES study.** (A) the first (PC1) and the second principle component (PC2) of Chinese samples with Singapore Genome Variation Project (SGVP) individuals. These Chinese samples included the tested individuals of the three Singapore Prospective Study Program cohorts and Singapore Chinese Eye Study.
(B) the first (PC1) and second principle component (PC2) of Chinese samples in our study.
(JPG)

Figure S3 **Principle component analysis of SiMES study.** (A) the first (PC1) and the second principle component (PC2) of Malay samples with Singapore Genome Variation Project (SGVP) individuals.
(B) the first (PC1) and second principle component (PC2) of Malay samples in our study.
(JPG)

Figure S4 **Principle component analysis of SINDI study.** (A) the first (PC1) and second principle component (PC2) of Indian samples with Singapore Genome Variation Project (SGVP) individuals.
(B) the first (PC1) and second principle component (PC2) of Indian samples in our study.
(C) the second (PC2) and third principle component (PC3) of Indian samples with SGVP individuals.
(D) the second (PC2) and third principle component (PC3) of Indian samples in our study.
(JPG)

Figure S5 **Genome-wide association scans in Chinese, Malays and Indians separately.** The –log_10_ of P-values (Y-axis) of combined Chinese (A), Malays (B) and Indians (C) are plotted against the genomic coordinates (X-axis). Only autosomal chromosomes are plotted.
(JPG)

Figure S6 **Q-Q plots of genome-wide association P-values of Chinese, Malays and Indians separately.** A, B and C are the Q-Q plots of combined Chinese, Malays and Indians respectively. The Y-axis represents the observed distribution of P-values while the X-axis represents the expected distribution.
(JPG)

Table S1 ***G6PC3* significant SNPs in Malays, Chinese, Indians and meta-analysis.** Chr, represents the chromosome number of the SNPs; BP, position; EA, effect allele; OA, other allele; N, sample size; EAF, effect allele frequency; Beta, linear regression coefficient; SE, standard error of Beta; Cohort, the population in which the statistic was measured. Among the cluster of SNPs, rs12603404 was genotyped in all cohorts, hence chosen as index SNP. Note that rs5036 was poorly imputed in SINDI (r2=0.12), so its result in Indians is not shown.
(DOCX)

Table S2 **Transferability of European established loci in Asian populations.** Chr, represents the chromosome number of the SNPs; Pos, position; EA, effect allele; OA, other allele; N, sample size; EAF, effect allele frequency; Beta, linear regression coefficient; SE, standard error of Beta; R_Fst, regional F_ST_; hap_ent, haplotype entropy. The literatures from which we extracted the reported effect are given in the source column. Whenever possible, we adopted the reported effect from Soranzo, et.al. Regional F_ST_ and haplotype entropy were only calculated for each population, instead of the meta-analysis.
(DOCX)

Table S3 **Association evidence of European established HbA_1C_ SNPs with moderate/severe DR.** Retinopathy was graded according to the modified Airlie House classification system. The moderate/severe diabetes retinopathy (DR) was defined as case: grade >=30; control: grade < 14. The odds ratios (OR) and 95% confidence interval (CI) were calculated from the beta coefficients of the logistic regression. P-values less than or equal to 0.05 are highlighted in bold.
(DOCX)

Table S4 **Association evidence of European established HbA_1C_ SNPs with any DR.** Retinopathy was graded according to the modified Airlie House classification system. Any DR case was defined as grade >=14, while control was defined as grade < 14. P-values less than or equal to 0.05 are highlighted in bold.
(DOCX)
