# Supplementary figures and images for: A Study Assessing the Association of Glycated Hemoglobin A1C (HbA1C) Associated Variants with HbA1C, Chronic Kidney Disease and Diabetic Retinopathy in Populations of Asian Ancestry
Source: PLoS One. 2013 Nov 7;8(11):e79767. doi: 10.1371/journal.pone.0079767 (PMC3820602; doi:10.1371/journal.pone.0079767)

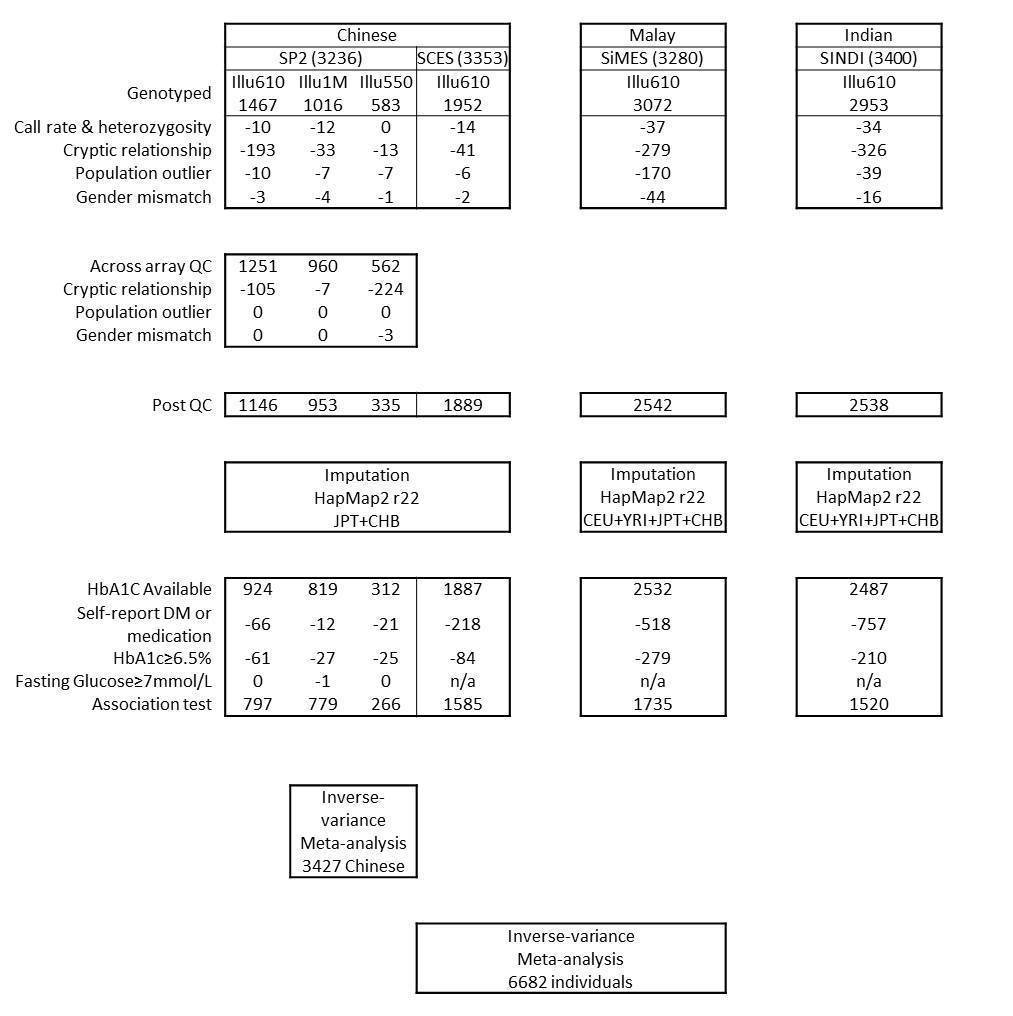

Supplement: Figure S1 — Flow chart of the GWAS meta-analysis. SP2, Singapore Prospective Study Program; SCES, Singapore Chinese Eye Study; SiMES, Singapore Malay Eye Study; SINDI, Singapore Indian Eye Study; Illu610, Illu1M and Illu550 are Illumina HumanHap 610 Quad, 1M Duo and 550 v3 array platforms respectively. (JPG) [file pone.0079767.s001.jpg]

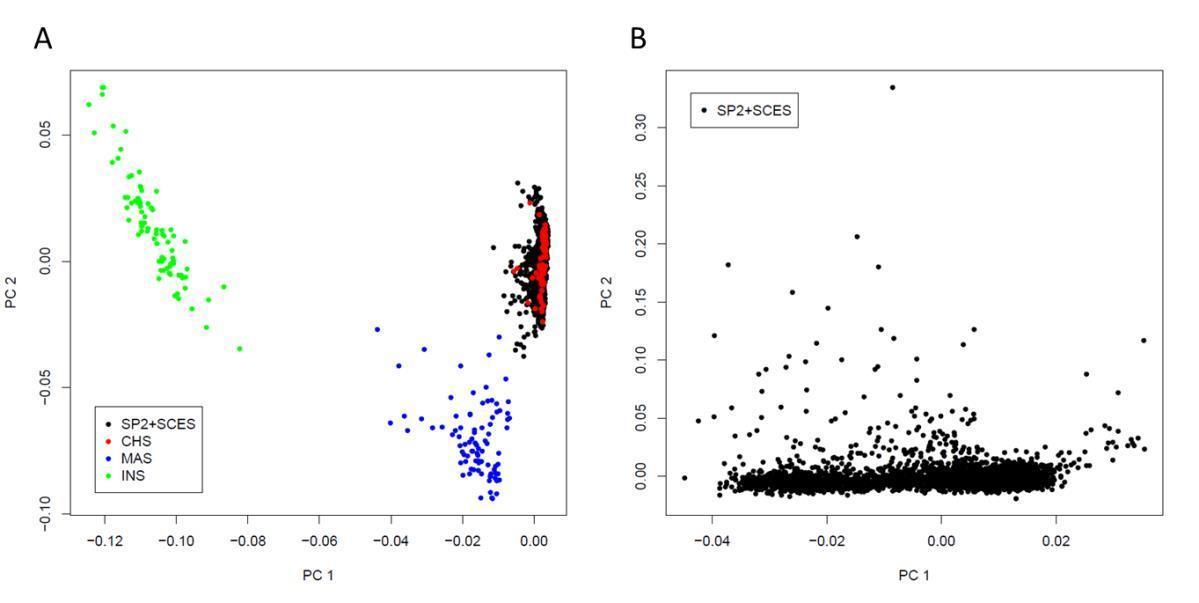

Supplement: Figure S2 — Principle component analysis of SP2 and SCES study. (A) the first (PC1) and the second principle component (PC2) of Chinese samples with Singapore Genome Variation Project (SGVP) individuals. These Chinese samples included the tested individuals of the three Singapore Prospective Study Program cohorts and Singapore Chinese Eye Study. (B) the first (PC1) and second principle component (PC2) of Chinese samples in our study. (JPG) [file pone.0079767.s002.jpg]

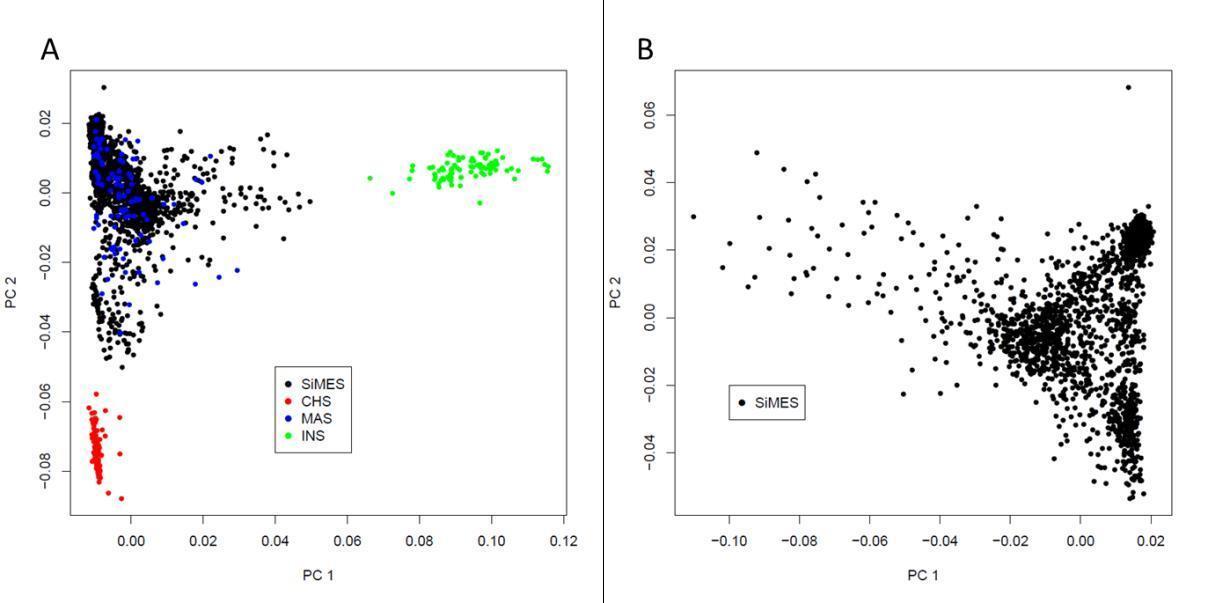

Supplement: Figure S3 — Principle component analysis of SiMES study. (A) the first (PC1) and the second principle component (PC2) of Malay samples with Singapore Genome Variation Project (SGVP) individuals. (B) the first (PC1) and second principle component (PC2) of Malay samples in our study. (JPG) [file pone.0079767.s003.jpg]

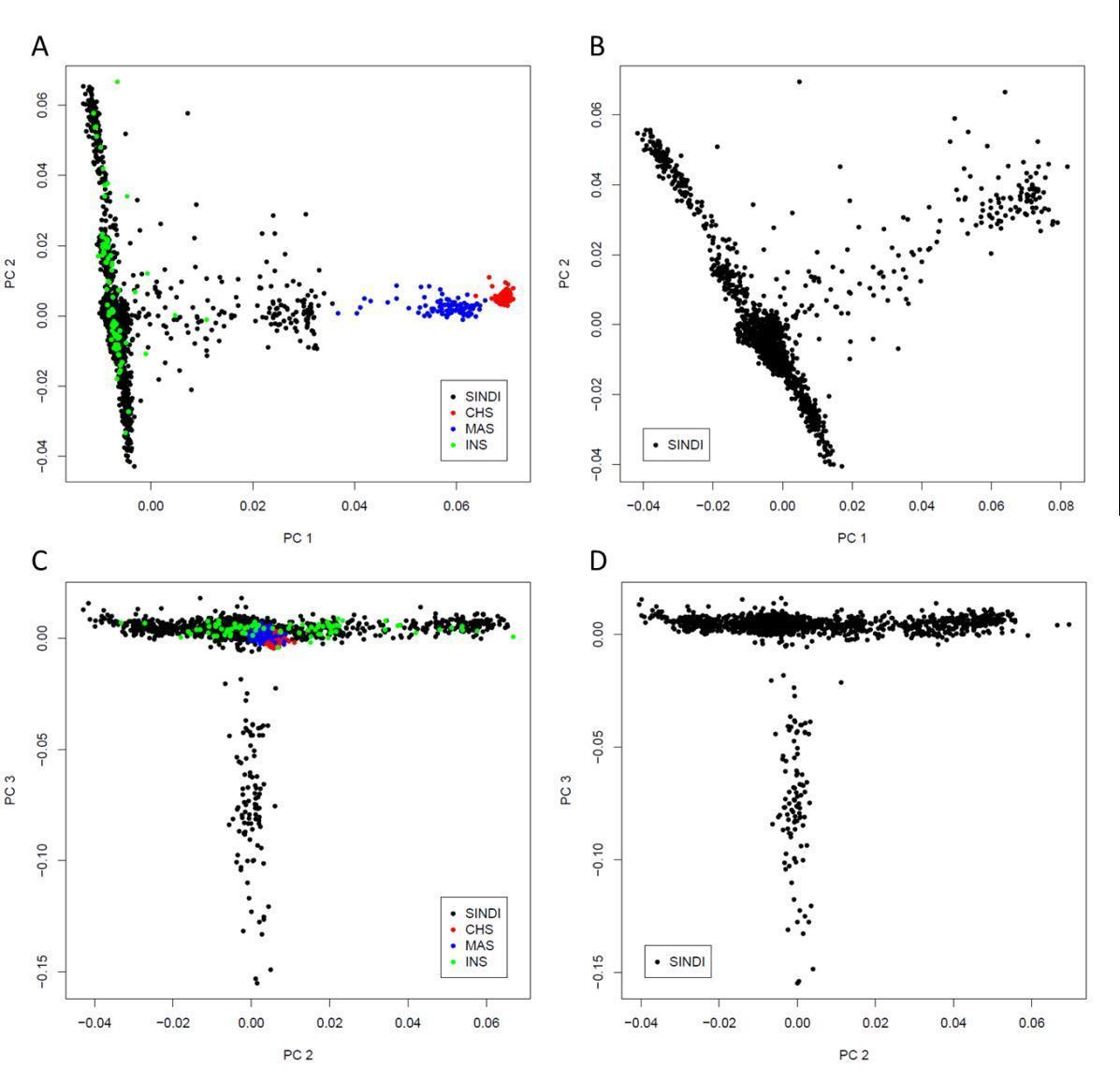

Supplement: Figure S4 — Principle component analysis of SINDI study. (A) the first (PC1) and second principle component (PC2) of Indian samples with Singapore Genome Variation Project (SGVP) individuals. (B) the first (PC1) and second principle component (PC2) of Indian samples in our study. (C) the second (PC2) and third principle component (PC3) of Indian samples with SGVP individuals. (D) the second (PC2) and third principle component (PC3) of Indian samples in our study. (JPG) [file pone.0079767.s004.jpg]

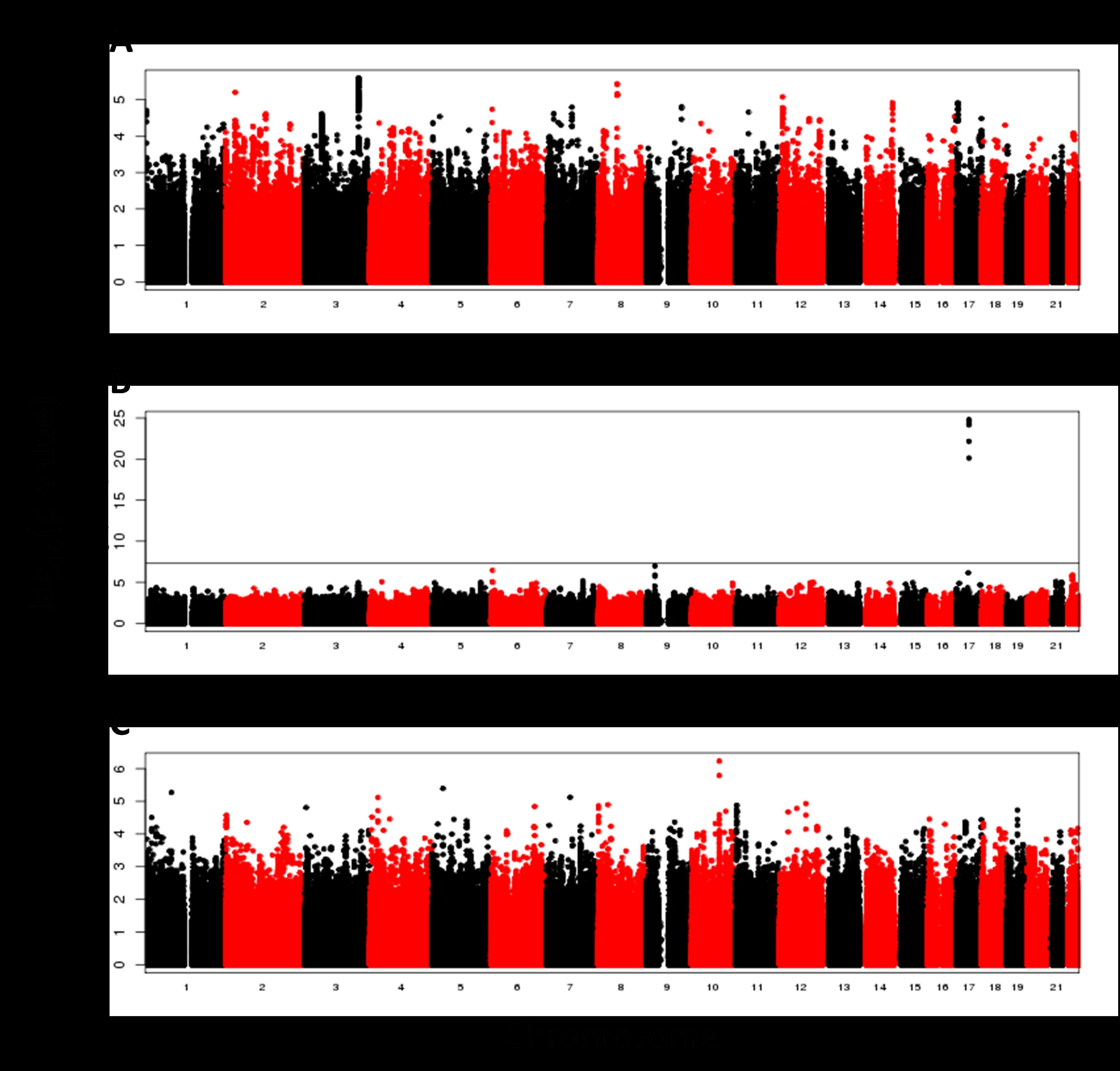

Supplement: Figure S5 — Genome-wide association scans in Chinese, Malays and Indians separately. The –log10 of P-values (Y-axis) of combined Chinese (A), Malays (B) and Indians (C) are plotted against the genomic coordinates (X-axis). Only autosomal chromosomes are plotted. (JPG) [file pone.0079767.s005.jpg]

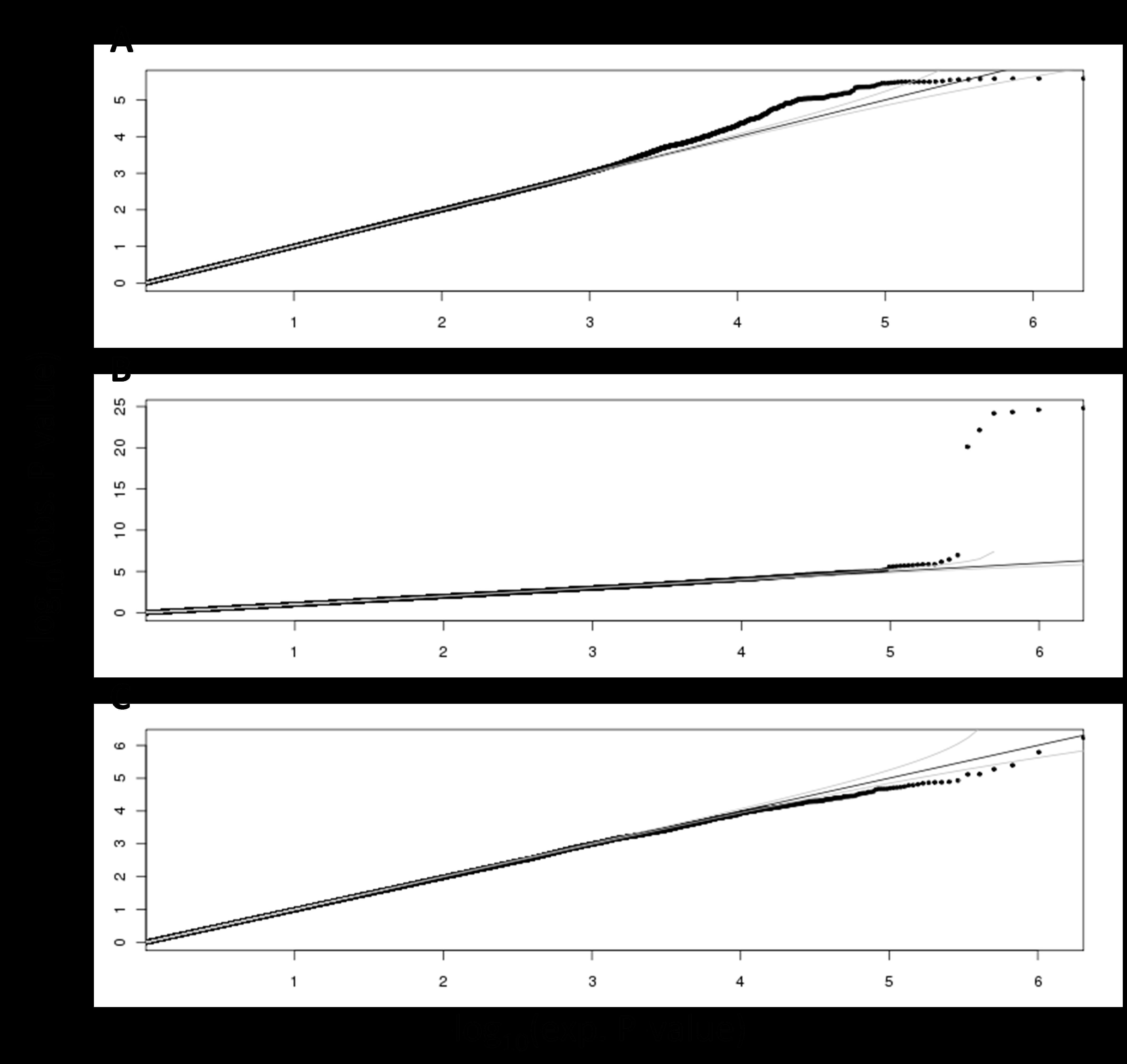

Supplement: Figure S6 — Q-Q plots of genome-wide association P-values of Chinese, Malays and Indians separately. A, B and C are the Q-Q plots of combined Chinese, Malays and Indians respectively. The Y-axis represents the observed distribution of P-values while the X-axis represents the expected distribution. (JPG) [file pone.0079767.s006.jpg]
