# Supplementary material for: A Study Assessing the Association of Glycated Hemoglobin A1C (HbA1C) Associated Variants with HbA1C, Chronic Kidney Disease and Diabetic Retinopathy in Populations of Asian Ancestry
Source: PLoS One. 2013 Nov 7;8(11):e79767. doi: 10.1371/journal.pone.0079767 (PMC3820602; doi:10.1371/journal.pone.0079767)
Supplement: Table S1 — G6PC3 significant SNPs in Malays, Chinese, Indians and meta-analysis. Chr, represents the chromosome number of the SNPs; BP, position; EA, effect allele; OA, other allele; N, sample size; EAF, effect allele frequency; Beta, linear regression coefficient; SE, standard error of Beta; Cohort, the population in which the statistic was measured. Among the cluster of SNPs, rs12603404 was genotyped in all cohorts, hence chosen as index SNP. Note that rs5036 was poorly imputed in SINDI (r2=0.12), so its result in Indians is not shown. (DOCX) [file pone.0079767.s007.docx]

| SNP | Chr | BP | EA | OA | N | EAF | Beta | SE | P-value | Cohort |
| --- | --- | --- | --- | --- | --- | --- | --- | --- | --- | --- |
| rs12603404 | 17 | 39,579,440 | A | G | 1735 | 0.03 | -0.330 | 0.034 | 3.89E-22 | Malay |
|  |  |  |  |  | 3427 | 0.05 | 0.006 | 0.018 | 7.26E-01 | Chinese |
|  |  |  |  |  | 1520 | 0.02 | -0.017 | 0.041 | 6.75E-01 | Indian |
|  |  |  |  |  | 6682 | 0.04 | -0.061 | 0.015 | 4.52E-05 | Meta-analysis |
| rs12601855 | 17 | 39,579,549 | G | A | 1735 | 0.03 | -0.354 | 0.035 | 4.33E-24 | Malay |
|  |  |  |  |  | 3427 | 0.05 | 0.007 | 0.018 | 7.22E-01 | Chinese |
|  |  |  |  |  | 1520 | 0.02 | -0.016 | 0.041 | 7.06E-01 | Indian |
|  |  |  |  |  | 6682 | 0.04 | -0.063 | 0.015 | 2.92E-05 | Meta-analysis |
| rs16940539 | 17 | 39,580,107 | G | C | 1735 | 0.03 | -0.356 | 0.035 | 3.16E-24 | Malay |
|  |  |  |  |  | 3427 | 0.05 | 0.007 | 0.018 | 7.22E-01 | Chinese |
|  |  |  |  |  | 1520 | 0.03 | -0.012 | 0.041 | 7.63E-01 | Indian |
|  |  |  |  |  | 6682 | 0.04 | -0.062 | 0.015 | 3.24E-05 | Meta-analysis |
| rs2285954 | 17 | 39,588,464 | G | T | 1735 | 0.03 | -0.359 | 0.035 | 1.64E-24 | Malay |
|  |  |  |  |  | 3427 | 0.05 | 0.007 | 0.018 | 7.04E-01 | Chinese |
|  |  |  |  |  | 1520 | 0.02 | -0.016 | 0.041 | 7.07E-01 | Indian |
|  |  |  |  |  | 6682 | 0.04 | -0.063 | 0.015 | 2.82E-05 | Meta-analysis |
| rs12602486 | 17 | 39,597,455 | G | T | 1735 | 0.03 | -0.362 | 0.035 | 1.04E-24 | Malay |
|  |  |  |  |  | 3426 | 0.05 | 0.007 | 0.018 | 6.87E-01 | Chinese |
|  |  |  |  |  | 1520 | 0.02 | -0.016 | 0.041 | 7.07E-01 | Indian |
|  |  |  |  |  | 6681 | 0.04 | -0.063 | 0.015 | 2.96E-05 | Meta-analysis |
| rs5036 | 17 | 39,694,471 | C | T | 1735 | 0.01 | -0.734 | 0.064 | 1.73E-20 | Malay |
|  |  |  |  |  | 778 | 0.06 | 0.041 | 0.038 | 2.85E-01 | Chinese |
|  |  |  |  |  | 2513 | 0.05 | -0.164 | 0.033 | 6.64E-07 | Meta-analysis |
