# Supplementary material for: A Study Assessing the Association of Glycated Hemoglobin A1C (HbA1C) Associated Variants with HbA1C, Chronic Kidney Disease and Diabetic Retinopathy in Populations of Asian Ancestry
Source: PLoS One. 2013 Nov 7;8(11):e79767. doi: 10.1371/journal.pone.0079767 (PMC3820602; doi:10.1371/journal.pone.0079767)
Supplement: Table S2 — Transferability of European established loci in Asian populations. Chr, represents the chromosome number of the SNPs; Pos, position; EA, effect allele; OA, other allele; N, sample size; EAF, effect allele frequency; Beta, linear regression coefficient; SE, standard error of Beta; R_Fst, regional FST; hap_ent, haplotype entropy. The literatures from which we extracted the reported effect are given in the source column. Whenever possible, we adopted the reported effect from Soranzo, et.al. Regional FST and haplotype entropy were only calculated for each population, instead of the meta-analysis. (DOCX) [file pone.0079767.s008.docx]

|  |  |  |  |  |  | EAF | Beta |  | | | | | |  |
| --- | --- | --- | --- | --- | --- | --- | --- | --- | --- | --- | --- | --- | --- | --- |
| SNP | Chr | BP | Gene | Source | EA | (European) | (European) | EAF | Beta(SE) | P-value | Power(%) | R_Fst | Hap_ent | Cohort |
| rs2779116 | 1 | 156,852,039 | SPTA1 | Soranzo, et.al | T | 0.27 | 0.024 | 0.43 | 0.016(0.008) | **3.72E-02** | **44.5** | **0.014** | **0.48** | Chinese |
|  |  |  |  |  |  |  |  | 0.46 | 0.012(0.013) | 3.40E-01 | 17.1 | 0.012 | 0.49 | Malay |
|  |  |  |  |  |  |  |  | 0.16 | -0.015(0.017) | 3.72E-01 | 13.9 | 0.017 | 0.20 | Indian |
|  |  |  |  |  |  |  |  | 0.40 | 0.011(0.006) | 6.99E-02 | 15.1 | **n/a** | **n/a** | Meta-analysis |
| rs1402837 | 2 | 169,465,600 | G6PC2 | Pare, et.al | T | 0.23 | 0.023 | 0.41 | 0.008(0.008) | 3.11E-01 | 14.8 | 0.039 | 0.61 | Chinese |
|  |  |  |  |  |  |  |  | 0.38 | 0.034(0.013) | **8.05E-03** | **77.2** | **0.048** | **0.62** | Malay |
|  |  |  |  |  |  |  |  | 0.22 | 0.018(0.015) | 2.28E-01 | 21.7 | 0.024 | 0.28 | Indian |
|  |  |  |  |  |  |  |  | 0.37 | 0.016(0.006) | **1.10E-02** | **18.5** | n/a | n/a | Meta-analysis |
| rs552976 | 2 | 169,499,684 | G6PC2,ABCB11 | Soranzo, et.al | G | 0.64 | 0.047 | 0.99 | -0.036(0.037) | 3.24E-01 | 18.3 | 0.045 | 0.88 | Chinese |
|  |  |  |  |  |  |  |  | 0.97 | 0.046(0.036) | 2.00E-01 | 22.9 | 0.050 | 0.90 | Malay |
|  |  |  |  |  |  |  |  | 0.83 | 0.014(0.017) | 3.88E-01 | 14.4 | 0.023 | 0.37 | Indian |
|  |  |  |  |  |  |  |  | 0.87 | 0.012(0.014) | 3.96E-01 | 6.7 | n/a | n/a | Meta-analysis |
| rs730497 | 7 | 44,190,246 | GCK | Pare, et.al | G | 0.83 | -0.030 | 0.80 | -0.039(0.01) | **8.57E-05** | **93.8** | **0.009** | **0.42** | Chinese |
|  |  |  |  |  |  |  |  | 0.88 | -0.059(0.02) | **2.97E-03** | **84.4** | **0.010** | **0.31** | Malay |
|  |  |  |  |  |  |  |  | 0.88 | -0.045(0.019) | **2.05E-02** | **63.4** | **0.012** | **0.22** | Indian |
|  |  |  |  |  |  |  |  | 0.83 | -0.043(0.008) | **8.14E-08** | **72.0** | n/a | n/a | Meta-analysis |
| rs1799884 | 7 | 44,195,593 | GCK | Soranzo, et.al | T | 0.18 | 0.038 | 0.20 | 0.039(0.01) | **7.49E-05** | **93.8** | **0.009** | **0.44** | Chinese |
|  |  |  |  |  |  |  |  | 0.11 | 0.061(0.02) | **2.16E-03** | **86.7** | **0.011** | **0.34** | Malay |
|  |  |  |  |  |  |  |  | 0.12 | 0.045(0.019) | **2.09E-02** | **61.5** | **0.009** | **0.25** | Indian |
|  |  |  |  |  |  |  |  | 0.17 | 0.044(0.008) | **5.62E-08** | **74.0** | n/a | n/a | Meta-analysis |
| rs6474359 | 8 | 41,668,351 | ANK1 | Soranzo, et.al | T | 0.97 | 0.058 | 0.97 | 0.003(0.022) | 8.80E-01 | 5.2 | 0.022 | 0.30 | Chinese |
|  |  |  |  |  |  |  |  | 0.98 | -0.003(0.052) | 9.53E-01 | 5.1 | 0.014 | 0.36 | Malay |
|  |  |  |  |  |  |  |  | 0.97 | 0.025(0.043) | 5.59E-01 | 10.7 | 0.007 | 0.16 | Indian |
|  |  |  |  |  |  |  |  | 0.97 | 0.007(0.018) | 7.23E-01 | 5.5 | **n/a** | **n/a** | Meta-analysis |
| rs4737009 | 8 | 41,749,562 | ANK1 | Soranzo, et.al | G | 0.76 | -0.027 | 0.51 | -0.01(0.01) | 3.11E-01 | 21.0 | 0.075 | 0.56 | Chinese |
|  |  |  |  |  |  |  |  | 0.60 | -0.011(0.016) | 5.03E-01 | 11.7 | 0.025 | 0.51 | Malay |
|  |  |  |  |  |  |  |  | 0.78 | -0.001(0.017) | 9.42E-01 | 5.1 | 0.005 | 0.20 | Indian |
|  |  |  |  |  |  |  |  | 0.58 | -0.008(0.007) | 2.59E-01 | 9.6 | **n/a** | **n/a** | Meta-analysis |
| rs13266634 | 8 | 118,253,964 | SLC30A8 | Pare, et.al | T | 0.30 | -0.019 | 0.47 | -0.018(0.008) | **2.13E-02** | **58.6** | **0.032** | **0.44** | Chinese |
|  |  |  |  |  |  |  |  | 0.43 | -0.032(0.013) | **1.23E-02** | **68.9** | **0.020** | **0.40** | Malay |
|  |  |  |  |  |  |  |  | 0.23 | -0.01(0.015) | 4.91E-01 | 11.3 | 0.008 | 0.25 | Indian |
|  |  |  |  |  |  |  |  | 0.42 | -0.02(0.006) | **1.03E-03** | **43.1** | n/a | n/a | Meta-analysis |
| rs7072268 | 10 | 70,769,919 | HK1 | Pare, et.al | T | 0.50 | 0.018 | 0.76 | 0.008(0.009) | 3.71E-01 | 14.3 | 0.045 | 0.72 | Chinese |
|  |  |  |  |  |  |  |  | 0.71 | 0.004(0.014) | 7.75E-01 | 6.4 | 0.046 | 0.82 | Malay |
|  |  |  |  |  |  |  |  | 0.43 | 0.013(0.013) | 3.13E-01 | 17.3 | 0.018 | 0.53 | Indian |
|  |  |  |  |  |  |  |  | 0.66 | 0.008(0.007) | 1.94E-01 | 11.7 | **n/a** | **n/a** | Meta-analysis |
| rs7903146 | 10 | 114,748,339 | TCF7L2 | Franklin, et. al. | T | 0.28 | 0.054 | 0.02 | -0.017(0.027) | 5.34E-01 | 8.1 | 0.112 | 0.54 | Chinese |
|  |  |  |  |  |  |  |  | 0.04 | -0.01(0.033) | 7.71E-01 | 6.1 | 0.070 | 0.51 | Malay |
|  |  |  |  |  |  |  |  | 0.27 | 3.2E-5(0.014) | 9.98E-01 | 5.0 | 0.009 | 0.25 | Indian |
|  |  |  |  |  |  |  |  | 0.19 | -0.004(0.012) | 7.11E-01 | 6.2 | **n/a** | **n/a** | Meta-analysis |
| rs1387153 | 11 | 92,313,476 | MTNR1B | Soranzo, et.al | T | 0.28 | 0.028 | 0.47 | 0.004(0.008) | 6.56E-01 | 7.5 | 0.035 | 0.75 | Chinese |
|  |  |  |  |  |  |  |  | 0.42 | 0.001(0.013) | 9.56E-01 | 5.1 | 0.035 | 0.82 | Malay |
|  |  |  |  |  |  |  |  | 0.37 | 0.005(0.013) | 7.15E-01 | 6.7 | 0.013 | 0.40 | Indian |
|  |  |  |  |  |  |  |  | 0.44 | 0.003(0.006) | 5.94E-01 | 6.1 | **n/a** | **n/a** | Meta-analysis |
| rs7998202 | 13 | 112,379,869 | ATP11A,TUBGCP3 | Soranzo, et.al | G | 0.14 | 0.031 | 0.06 | 0.022(0.016) | 1.71E-01 | 22.5 | 0.028 | 0.48 | Chinese |
|  |  |  |  |  |  |  |  | 0.08 | 0.017(0.024) | 4.67E-01 | 11.0 | 0.025 | 0.55 | Malay |
|  |  |  |  |  |  |  |  | 0.09 | 0.027(0.022) | 2.13E-01 | 22.9 | 0.013 | 0.28 | Indian |
|  |  |  |  |  |  |  |  | 0.08 | 0.022(0.011) | **4.93E-02** | **17.1** | n/a | n/a | Meta-analysis |
| rs1046896 | 17 | 78,278,822 | FN3K | Soranzo, et.al | T | 0.31 | 0.035 | 0.51 | 0.028(0.008) | **3.20E-04** | **89.6** | **0.023** | **0.19** | Chinese |
|  |  |  |  |  |  |  |  | 0.46 | 0.022(0.013) | 8.05E-02 | 36.7 | 0.013 | 0.16 | Malay |
|  |  |  |  |  |  |  |  | 0.37 | 0.046(0.013) | **4.05E-04** | **93.8** | **0.011** | **0.17** | Indian |
|  |  |  |  |  |  |  |  | 0.47 | 0.031(0.006) | **2.41E-07** | **73.7** | n/a | n/a | Meta-analysis |
| rs855791 | 22 | 35,792,882 | TMPRSS6 | Soranzo, et.al | G | 0.58 | -0.027 | 0.46 | -0.028(0.008) | **3.82E-04** | **89.3** | **0.017** | **0.75** | Chinese |
|  |  |  |  |  |  |  |  | 0.43 | -0.008(0.013) | 5.22E-01 | 8.4 | 0.025 | 0.68 | Malay |
|  |  |  |  |  |  |  |  | 0.50 | -0.019(0.013) | 1.21E-01 | 35.0 | 0.019 | 0.48 | Indian |
|  |  |  |  |  |  |  |  | 0.46 | -0.022(0.006) | **2.35E-04** | **50.2** | n/a | n/a | Meta-analysis |
| rs16926246 | 10 | 70,763,398 | HK1 | Soranzo, et.al | T | 0.10 | -0.089 | 0.05 | 0.028(0.033) | 3.96E-01 | 16.1 | 0.019 | 0.48 | Indian |
