# Supplementary material for: A Study Assessing the Association of Glycated Hemoglobin A1C (HbA1C) Associated Variants with HbA1C, Chronic Kidney Disease and Diabetic Retinopathy in Populations of Asian Ancestry
Source: PLoS One. 2013 Nov 7;8(11):e79767. doi: 10.1371/journal.pone.0079767 (PMC3820602; doi:10.1371/journal.pone.0079767)
Supplement: Table S3 — Association evidence of European established HbA1C SNPs with moderate/severe DR. Retinopathy was graded according to the modified Airlie House classification system. The moderate/severe diabetes retinopathy (DR) was defined as case: grade >=30; control: grade < 14. The odds ratios (OR) and 95% confidence interval (CI) were calculated from the beta coefficients of the logistic regression. P-values less than or equal to 0.05 are highlighted in bold. (DOCX) [file pone.0079767.s009.docx]

|  |  |  |  |  |  |  | Combined | |  | T2D Cases | |  | T2D Controls | |
| --- | --- | --- | --- | --- | --- | --- | --- | --- | --- | --- | --- | --- | --- | --- |
| SNP | Chr | BP | Gene | EA | OA | EAF | OR[0.95CI] | P-value |  | OR[0.95CI] | P-value |  | OR[0.95CI] | P-value |
| rs2779116 | 1 | 156,852,039 | SPTA1 | T | C | 0.40 | 1.06 [0.89, 1.28] | 5.11E-01 |  | 1.04 [0.86, 1.26] | 6.78E-01 |  | 1.42 [0.70, 2.87] | 3.27E-01 |
| rs1402837 | 2 | 169,465,600 | G6PC2 | T | C | 0.35 | 0.99 [0.83, 1.18] | 9.36E-01 |  | 1.01 [0.85, 1.21] | 9.05E-01 |  | 0.76 [0.38, 1.52] | 4.33E-01 |
| rs552976 | 2 | 169,499,684 | G6PC2,ABCB11 | A | G | 0.14 | 1.09 [0.83, 1.41] | 5.47E-01 |  | 1.11 [0.85, 1.46] | 4.34E-01 |  | 0.52 [0.12, 2.17] | 3.71E-01 |
| rs730497 | 7 | 44,190,246 | GCK | A | G | 0.17 | 1.10 [0.87, 1.38] | 4.18E-01 |  | 1.10 [0.87, 1.40] | 4.21E-01 |  | 1.06 [0.43, 2.62] | 9.02E-01 |
| rs1799884 | 7 | 44,195,593 | GCK | T | C | 0.17 | 1.11 [0.88, 1.40] | 3.76E-01 |  | 1.11 [0.88, 1.41] | 3.78E-01 |  | 1.06 [0.43, 2.62] | 9.02E-01 |
| rs6474359 | 8 | 41,668,351 | ANK1 | T | C | 0.97 | 1.24 [0.69, 2.23] | 4.64E-01 |  | 1.14 [0.62, 2.11] | 6.66E-01 |  | 2.80 [0.41, 18.96] | 2.91E-01 |
| rs4737009 | 8 | 41,749,562 | ANK1 | A | G | 0.44 | 0.97 [0.78, 1.19] | 7.44E-01 |  | 0.99 [0.79, 1.23] | 9.16E-01 |  | 0.71 [0.32, 1.57] | 4.03E-01 |
| rs13266634 | 8 | 118,253,964 | SLC30A8 | T | C | 0.41 | 0.95 [0.80, 1.13] | 5.95E-01 |  | 1.00 [0.83, 1.19] | 9.64E-01 |  | 0.51 [0.25, 1.01] | 5.19E-02 |
| rs7072268 | 10 | 70,769,919 | HK1 | T | C | 0.64 | 1.07 [0.91, 1.26] | 4.19E-01 |  | 1.09 [0.92, 1.29] | 3.30E-01 |  | 0.80 [0.39, 1.64] | 5.41E-01 |
| rs7903146 | 10 | 114,748,339 | TCF7L2 | T | C | 0.21 | 1.21 [0.98, 1.50] | 7.89E-02 |  | 1.23 [0.99, 1.53] | 6.76E-02 |  | 0.86 [0.26, 2.91] | 8.14E-01 |
| rs1387153 | 11 | 92,313,476 | MTNR1B | T | C | 0.43 | 1.08 [0.92, 1.27] | 3.28E-01 |  | 1.11 [0.94, 1.31] | 2.18E-01 |  | 0.72 [0.37, 1.42] | 3.47E-01 |
| rs7998202 | 13 | 112,379,869 | ATP11A,TUBGCP3 | A | G | 0.93 | 1.04 [0.79, 1.37] | 7.64E-01 |  | 1.03 [0.78, 1.37] | 8.26E-01 |  | 1.30 [0.36, 4.64] | 6.91E-01 |
| rs1046896 | 17 | 78,278,822 | FN3K | T | C | 0.46 | 1.07 [0.91, 1.25] | 4.23E-01 |  | 1.07 [0.91, 1.25] | 4.41E-01 |  | 1.08 [0.55, 2.11] | 8.23E-01 |
| rs855791 | 22 | 35,792,882 | TMPRSS6 | A | G | 0.53 | 1.05 [0.90, 1.23] | 5.17E-01 |  | 1.03 [0.88, 1.21] | 7.25E-01 |  | 1.55 [0.80, 2.99] | 1.96E-01 |
| rs16926246 | 10 | 70,763,398 | HK1 | T | C | 0.04 | 0.54 [0.30, 0.97] | **3.88E-02** |  | 0.55 [0.30, 1.02] | 5.68E-02 |  | 0.34 [0.03, 3.66] | 3.72E-01 |
