# Supplementary material for: A Study Assessing the Association of Glycated Hemoglobin A1C (HbA1C) Associated Variants with HbA1C, Chronic Kidney Disease and Diabetic Retinopathy in Populations of Asian Ancestry
Source: PLoS One. 2013 Nov 7;8(11):e79767. doi: 10.1371/journal.pone.0079767 (PMC3820602; doi:10.1371/journal.pone.0079767)
Supplement: Table S4 — Association evidence of European established HbA1C SNPs with any DR. Retinopathy was graded according to the modified Airlie House classification system. Any DR case was defined as grade >=14, while control was defined as grade < 14. P-values less than or equal to 0.05 are highlighted in bold. (DOCX) [file pone.0079767.s010.docx]

|  |  |  |  |  |  |  | Combined | |  | T2D Cases | |  | T2D Controls | |
| --- | --- | --- | --- | --- | --- | --- | --- | --- | --- | --- | --- | --- | --- | --- |
| SNP | Chr | BP | Gene | EA | OA | EAF | OR[0.95CI] | P-value |  | OR[0.95CI] | P-value |  | OR[0.95CI] | P-value |
| rs2779116 | 1 | 156,852,039 | SPTA1 | T | C | 0.41 | 0.98 [0.86, 1.10] | 6.94E-01 |  | 0.99 [0.85, 1.16] | 9.32E-01 |  | 0.95 [0.78, 1.16] | 6.00E-01 |
| rs1402837 | 2 | 169,465,600 | G6PC2 | T | C | 0.36 | 1.06 [0.94, 1.20] | 3.27E-01 |  | 1.05 [0.91, 1.22] | 4.98E-01 |  | 1.08 [0.88, 1.31] | 4.65E-01 |
| rs552976 | 2 | 169,499,684 | G6PC2,ABCB11 | A | G | 0.12 | 1.06 [0.87, 1.30] | 5.61E-01 |  | 1.16 [0.93, 1.46] | 1.95E-01 |  | 0.76 [0.49, 1.17] | 2.18E-01 |
| rs730497 | 7 | 44,190,246 | GCK | A | G | 0.16 | 1.09 [0.93, 1.28] | 2.95E-01 |  | 1.08 [0.88, 1.32] | 4.55E-01 |  | 1.11 [0.85, 1.44] | 4.53E-01 |
| rs1799884 | 7 | 44,195,593 | GCK | T | C | 0.16 | 1.11 [0.94, 1.30] | 2.10E-01 |  | 1.09 [0.90, 1.34] | 3.76E-01 |  | 1.13 [0.87, 1.48] | 3.64E-01 |
| rs6474359 | 8 | 41,668,351 | ANK1 | T | C | 0.97 | 1.39 [0.94, 2.06] | 9.59E-02 |  | 1.13 [0.67, 1.89] | 6.46E-01 |  | 1.84 [1.01, 3.33] | **4.47E-02** |
| rs4737009 | 8 | 41,749,562 | ANK1 | A | G | 0.42 | 0.93 [0.81, 1.08] | 3.63E-01 |  | 0.99 [0.82, 1.19] | 9.14E-01 |  | 0.86 [0.68, 1.08] | 1.88E-01 |
| rs13266634 | 8 | 118,253,964 | SLC30A8 | T | C | 0.42 | 0.92 [0.82, 1.04] | 1.81E-01 |  | 1.02 [0.88, 1.19] | 7.59E-01 |  | 0.77 [0.63, 0.94] | **9.35E-03** |
| rs7072268 | 10 | 70,769,919 | HK1 | T | C | 0.65 | 1.03 [0.91, 1.15] | 6.81E-01 |  | 1.03 [0.90, 1.19] | 6.48E-01 |  | 1.01 [0.82, 1.24] | 9.51E-01 |
| rs7903146 | 10 | 114,748,339 | TCF7L2 | T | C | 0.19 | 1.09 [0.92, 1.29] | 3.09E-01 |  | 1.18 [0.98, 1.43] | 7.56E-02 |  | 0.79 [0.54, 1.14] | 2.05E-01 |
| rs1387153 | 11 | 92,313,476 | MTNR1B | T | C | 0.43 | 1.10 [0.98, 1.23] | 1.08E-01 |  | 1.15 [1.00, 1.32] | **4.30E-02** |  | 0.99 [0.82, 1.20] | 9.42E-01 |
| rs7998202 | 13 | 112,379,869 | ATP11A,TUBGCP3 | A | G | 0.93 | 0.96 [0.79, 1.17] | 6.63E-01 |  | 0.95 [0.75, 1.20] | 6.87E-01 |  | 0.97 [0.67, 1.40] | 8.59E-01 |
| rs1046896 | 17 | 78,278,822 | FN3K | T | C | 0.46 | 1.03 [0.92, 1.15] | 6.57E-01 |  | 1.02 [0.89, 1.16] | 8.23E-01 |  | 1.04 [0.86, 1.27] | 6.53E-01 |
| rs855791 | 22 | 35,792,882 | TMPRSS6 | A | G | 0.54 | 1.08 [0.96, 1.20] | 1.94E-01 |  | 1.00 [0.87, 1.15] | 9.76E-01 |  | 1.24 [1.02, 1.50] | **2.81E-02** |
| rs16926246 | 10 | 70,763,398 | HK1 | T | C | 0.05 | 0.62 [0.38, 0.99] | **4.56E-02** |  | 0.62 [0.37, 1.06] | 8.21E-02 |  | 0.59 [0.21, 1.67] | 3.22E-01 |
